# Estimation of Low Quantity Genes: A Hierarchical Model for Analyzing Censored Quantitative Real-Time PCR Data

**DOI:** 10.1371/journal.pone.0064900

**Published:** 2013-05-31

**Authors:** Tim C. Boyer, Tim Hanson, Randall S. Singer

**Affiliations:** 1 Division of Environmental Health Sciences, School of Public Health, University of Minnesota, Minneapolis, Minnesota, United States of America; 2 Department of Statistics, University of South Carolina, Columbia, South Carolina, United States of America; 3 Department of Veterinary and Biomedical Sciences, University of Minnesota, St. Paul, Minnesota, United States of America; Albert Einstein College of Medicine, United States of America

## Abstract

Analysis of gene quantities measured by quantitative real-time PCR (qPCR) can be complicated by observations that are below the limit of quantification (LOQ) of the assay. A hierarchical model estimated using MCMC methods was developed to analyze qPCR data of genes with observations that fall below the LOQ (censored observations). Simulated datasets with moderate to very high levels of censoring were used to assess the performance of the model; model results were compared to approaches that replace censored observations with a value on the log scale approximating zero or with values ranging from one to the LOQ of ten gene copies. The model was also compared to a Tobit regression model. Finally, all approaches for handling censored observations were evaluated with DNA extracted from samples that were spiked with known quantities of the antibiotic resistance gene *tetL*. For the simulated datasets, the model outperformed substitution of all values from 1–10 under all censoring scenarios in terms of bias, mean square error, and coverage of 95% confidence intervals for regression parameters. The model performed as well or better than substitution of a value approximating zero under two censoring scenarios (approximately 57% and 79% censored values). The model also performed as well or better than Tobit regression in two of three censoring scenarios (approximately 79% and 93% censored values). Under the levels of censoring present in the three scenarios of this study, substitution of any values greater than 0 produced the least accurate results. When applied to data produced from spiked samples, the model produced the lowest mean square error of the three approaches. This model provides a good alternative for analyzing large amounts of left-censored qPCR data when the goal is estimation of population parameters. The flexibility of this approach can accommodate complex study designs such as longitudinal studies.

## Introduction

Quantitative real-time PCR (qPCR) has become an important molecular tool in the life sciences for its ability to both detect and quantify minute amounts of target nucleic acid present in a sample. Quantification of nucleic acid is made possible through real-time measurement of a fluorescence signal that accumulates as the PCR target amplifies. The fractional cycle in which the signal crosses a threshold above the background level (Cq) is the unit of analysis of qPCR assays [1]. There is a theoretical log-linear relationship between the Cq and the starting concentration of the target nucleic acid sequence. The starting concentration of target nucleic acid sequences in unknown samples can be estimated by comparing unknown Cq’s to a calibration curve made up of Cq’s from samples of known quantity. This approach is often referred to as absolute quantification. In practice, the range of quantities in which this log-linear relationship holds true is limited, usually spanning 3 to 6 orders of magnitude, and is referred to as the linear dynamic range. The linear dynamic range of a qPCR assay defines the limits of quantification (LOQ) of that assay. Sample quantities above or below these limits cannot be reliably estimated. However, some qPCR applications, such as quantification of genetically modified organisms in food, require measurement of gene quantities in samples with concentrations near or below the LOQ [2].

Measurement of genes near the LOQ can present difficulties and is an active area of research [2]–[9]. It is standard practice for the same sample of DNA to be run multiple times (i.e. technical replicates) as a way to assess intra-assay variation. For samples with very low quantities of the target gene, it is common to have one or more of the sample’s technical replicates produce no signal or produce an estimate that is below the LOQ. A qPCR reaction with no sign of target amplification could indicate that the gene is truly absent from the sample or that the gene of interest was present in the reaction but failed to amplify above the threshold level of fluorescence by the last cycle of the reaction. Alternatively, no signal could result when the gene of interest was present in the sample but in such a low concentration that the probability of including at least one copy of the gene in the small amount of template added to the reaction was very low [1], [9]–[11]. In these situations, the number of technical replicates can be increased to improve the probability of detecting the gene of interest. However, increasing replicates will improve estimates of gene quantity only if some of the replicates are above the LOQ. For large studies with many samples, increasing the number of replicates is not always feasible.

When an analytical method is unable to distinguish true negative samples from samples that are positive at a low level, those samples are censored. Common methods for dealing with censored data are to delete the censored observations prior to analysis or to substitute some value for the censored samples, such as a fraction of the detection limit, or zero [9], [12], [13]. All of these methods have been shown to produce varying degrees of bias, depending on the level of censoring present in the data [14]–[16]. One qPCR study recommended that substituting zero for censored observations will produce less biased estimates than deletion of censored observations [9].

Many studies that have investigated the performance of qPCR near the limits of quantification and detection have focused at the level of individual samples and their replicates. However, for some applications, qPCR is used to quantify nucleic acid sequences for the purpose of estimating population level parameters. For example, in epidemiologic studies of antibiotic resistance, qPCR has been used to estimate the association between antibiotic resistance gene quantities in bacterial community DNA and various risk factors [17]–[25]. In these situations it is common to standardize the qPCR measurements either by back-calculating the gene quantity per qPCR reaction to quantity per sample volume or by reporting the gene quantity relative to a reference gene such as 16S rRNA that is used as a proxy for total bacterial quantity [26], [27]. In both cases the data are often log transformed to make a skewed distribution somewhat symmetric and stabilize the variance prior to statistical analysis. In this situation, substitution of zero will result in a log transformed value that is undefined and some arbitrarily low number on the log scale must be substituted.

The topic of censored observations in analytical methods has been covered in many fields. In general, simple substitution methods or exclusion of censored observations has the potential to bias results and it has been recommended that those methods should never be used [14−16], [28], . Alternative approaches have been proposed, such as parametric methods that randomly impute single values for censored observations by maximum likelihood estimation or regression on order statistics [14]. However, these methods are generally not adequate for epidemiological studies where the objective is to relate the quantity of a gene to risk factors, especially when the data come from more complex study designs such as longitudinal or clustered data [15].

Another option to accommodate censored data is to use a Tobit regression approach where it is assumed that the censored and observed data arise from the same underlying normal distribution and the regression parameters are estimated by maximum likelihood [15], [29]. A more flexible alternative is to create a hierarchical model that explicitly includes the censoring in the likelihood function and can be estimated using MCMC or other estimation algorithms [15], [28], [30]. The MCMC approach works by iteratively imputing the censored values, using the imputed values and observed data in a regression to obtain parameter estimates, and then using the parameter estimates to randomly impute new values for the censored data. Over many iterations, distributions of the missing values are created. This approach also permits flexibility in the data structure such as repeated measurements from the same sample.

The objective of this study is to develop a hierarchical model for analyzing censored qPCR data. The performance of the model was compared to substitution of fixed values for censored observations and Tobit regression using simulated data representing gene quantities in community bacterial DNA. We hypothesized that this model would produce more accurate estimates of regression population parameters than linear regression performed on simulated data where censored values are substituted with a single value. We also tested the model with antibiotic resistance gene quantities extracted from samples into which the gene had been spiked, allowing us to know the actual quantity per sample.

## Materials and Methods

### Rationale for Model Development

Many applications of qPCR measure bacterial genes in a cultivation-independent manner that permits estimation of gene quantity in the entire bacterial community of a sample. This approach requires extraction of community DNA from a sample such as soil or feces to yield a volume of DNA in solution. A small proportion of that DNA solution is then added to each replicate qPCR reaction. Therefore, the output of the qPCR reaction is gene quantity per volume of template added to the reaction. The model developed for this study estimated the unknown total log_10_ gene quantity per gram of sample from the observed gene quantities in the individual qPCR replicates, some of which were censored.

For this study, it was assumed that DNA extraction yielded 1000 µl of DNA solution for every sample *j*. From that total volume of extracted DNA, it was assumed that 5 µl of template DNA was added to each of three replicate qPCR reactions. The three 5 µl aliquots *x_j1_, x_j2_, x_j3_* of template and the remaining 985 µl (*x_j4_*) were assumed to follow a Multinomial*(n_j_, p_j_)* distribution, where *n_j_* equals the unknown quantity of the gene of interest in the entire sample *j* and *p_j_* is a vector of probabilities corresponding to the proportions of sample added to each of the triplicate reactions and the unused sample (e.g. *p* = 0.005, 0.005, 0.005, 0.985). Since the probabilities corresponding to the proportions of sample added to the sample aliquots are very small relative to the unused sample probability the model is well-approximated by “sampling with replacement” and we may consider the *x_i1_, x_i2_, x_i3_* as independent binomial (*n_j_, p_j_*) for j = 1,2,3. Furthermore, for moderate *n_j_p_j_* – which we have here – the binomial is well-approximated by the Poisson, yielding the following hierarchical model.
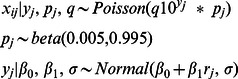
where *x_ij_* is the observed gene quantity of replicate *i* from sample *j*. The estimated mean of the replicates for sample *j* approximates the mean of a binomial distribution n_j_*p_j_ where *n_j_* is the unknown gene quantity of the entire volume of extracted DNA. The Poisson approximation to the binomial improves the stability of the model because there are fewer parameters to estimate. The unknown quantity (*n_j_*) is the product of a correction factor (*q*) for DNA extraction efficiency and the unknown log_10_ gene quantity (*y_j_*) in the entire sample. All DNA extraction protocols yield less than 100% of the DNA present in a sample due to incomplete cell lysis and volume losses during the extraction process [31]. Extraction efficiency varies by extraction protocol and the type of sample from which the DNA is extracted. Here the DNA extraction efficiency is assumed to be fixed at 65%. This 65% assumption is based on validation experiments of the DNA extraction method used by our laboratory (data not shown). If the extraction efficiency is unknown, a probability distribution can be assigned to the efficiency to reflect that uncertainty. In addition, it is assumed here that the original samples from which the DNA was extracted were all one gram. For samples of varying starting weight, an additional correction factor can be included to adjust for these variations. The probability of including a gene copy in the 5 µl added to a single qPCR reaction (*p_j_*) is assumed to follow a beta distribution centered around 0.005 to allow for extra-binomial variation in the observed data. The unknown log_10_ gene quantity (*y_j_*) is assumed to follow a normal distribution with a mean estimated by the regression equation *β0*+ β1*riskfactor, where the generic risk factor is a binary variable (1 or 0).

To estimate the quantities of censored observations, the censored observations (*x_ij_*) are expressed in terms of the true copy number (*z_ij_*). It was determined from the linear range of the calibration curve and dilution experiments that the LOQ for the genes studied by our laboratory is 10 gene copies per reaction (data not shown). Therefore, a LOQ of 10 copies per qPCR reaction was used for these simulations. The censored observations are expressed as the observed value if it is greater than or equal to 10, or ranging from 0 to 9 if censored:
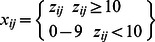
(1)


For uncensored data, the likelihood is:
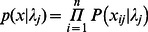
(2)where P is the Poisson probability density function (pdf), *λ_j_* is the sample mean, and *n* is the number of observations. Values for the censored observations are randomly sampled from a Poisson distribution, constrained from 0 to 9 gene copies, and the imputed and observed data together are used to estimate the parameters of the model (See Additional File 1 for code). The estimated parameters are then used to iteratively impute new values for the censored observations and then re-estimate the model parameters. A censored observation with a large estimated λ is more likely to be assigned a value closer to 9 on the following iteration than a censored observation with a very small estimated λ. After a number of iterations, posterior distributions of the parameters and censored observations are obtained.

### Simulation of Data

The performance of the model was assessed by testing it using simulated qPCR data. Datasets of 500 samples were generated representing log_10_ gene quantity per gram of sample. Each simulated dataset was generated assuming a normal distribution using three different mean values to test the model on datasets with three different degrees of censoring (Table 1). All datasets had a standard deviation of 1.5, and an indicator variable was used to randomly assign a binary risk factor to approximately 50% of the samples in each dataset resulting in gene copy numbers that were on average 0.5 log_10_ copies higher in the group with the risk factor than the group without the risk factor. Because the simulations were repeated using three different mean values on the log_10_ scale, the risk factor effect of 0.5 log_10_ copies ranged from approximately 21 gene copies to 2100 copies per sample. The risk factor value of 0.5 was selected as typical of statistically significant coefficients that we have observed in regression models of real qPCR data (data not shown). In addition, we simulated datasets with an intercept of 3 log_10_ gene copies, a standard deviation of 1.5 and a risk factor value of 0.1 to represent the lower bound of significant regression coefficients that we have observed in our real qPCR data.

**Table 1:** Parameter values used to generate simulated data of log_10_ gene copies per gram of sample.

Scenario	True Parameters	Mean (range) percent of censored observations	Mean number of censored observations per sample (0, 1, 2, 3)
1	β0 = 3, β1 = 0.5, σ = 1.5	57% (51%–62%)	189, 27, 26, 256
2	β0 = 2, β1 = 0.5, σ = 1.5	79% (75%–86%)	84, 18, 20, 378
3	β0 = 1, β1 = 0.5, σ = 1.5	93% (89%–96%)	27, 8, 10, 455

The simulated samples of log_10_ gene quantity per gram were transformed to gene quantity per gram and multiplied by 0.65 for assumed losses due to extraction efficiency to yield gene quantity per 1000 µl of DNA extract. To obtain the gene quantity added to each of the triplicate qPCR wells per sample, three 5 µl random samples were drawn from the simulated 1000 µl of DNA extract using a Dirichlet-multinomial distribution, a multivariate generalization of the beta-binomial distribution. The copy number per 1000 µl was used as the size parameter (*n*) and the four probability parameters each followed a beta distribution centered around 0.005, 0.005, 0.005, and 0.985 respectively. The probability parameters were allowed to follow beta distributions to produce sample triplicates that are more variable than what would be produced if the triplicates followed a binomial distribution with a fixed value for *p_j_*. Based on our experience with real qPCR data, this extra-binomial variation yielded simulated sample triplicates that more closely resembled reality than simulations assuming a binomial variance. This process produced three simulated observations per sample of gene quantity per 5 µl of DNA resulting in a dataset comprised of 1500 total observations for 500 samples. An LOQ of 10 copies was used as the censoring limit. All simulated observations with values less than 10 were set to missing so they could be estimated by the model. The simulated data were generated using R version 2.12.2 [32].

The model was run using WinBUGS (version 1.4), called from R with the R2Winbugs package, on 500 simulated datasets for each parameter combination using non-informative normal priors for *β*0 and *β*1 and a non-informative gamma prior for the inverse of *σ* [33], [34]. The model was run for 5000 iterations with a burn-in of 1000 iterations. Preliminary runs were made using three MCMC chains each with different initial parameter values. History plots, Gelman-Rubin statistic plots, and autocorrelation plots (not shown) of the regression parameters were examined to confirm model convergence. Afterward, one chain was run per dataset to speed the simulation. The R and WinBUGS code can be found in Additional File 1.

The posterior means of *β0*, *β1*, and *σ* for each iteration were stored. For every parameter the bias, mean square error (MSE), and proportion of 95% credible intervals that included the true values of their respective parameters were calculated [35],
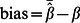
(3)

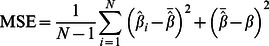
(4)


For comparison with the hierarchical model, 12 additional simulations were performed each on 500 datasets generated in the same way as described above. For one of the 12 simulations, censored observations were unchanged and Tobit regression was performed using the censReg function of the R package censReg. For the other 12 simulations censored observations were replaced with 0 or integers ranging from 1 to 10 gene copies. Triplicate observations were averaged and back-calculated to log_10_ gene quantity. For situations where all triplicates were below the LOQ, substitution of zero resulted in an average gene quantity that was undefined when transformed to log_10_ copies per gram of sample. The minimum non-zero concentration in a sample is 1 gene copy per gram or 0.00325log_10_ gene copies. For samples with three censored triplicates, a value of 0.00325log_10_ was substituted for the log transformed sample average to approximate a sample where the gene of interest is not detected in any of the replicates. The average log_10_ gene quantity of each sample was used as the dependent variable in a linear regression with the generic risk factor as the independent variable. For each of the 12 substitution scenarios and the Tobit regression scenario the bias and MSE were calculated for *β0*, *β1*, and percent coverage of 95% confidence intervals were calculated for *β0* and *β1*.

### Model Evaluation with Spiked Samples

The hierarchical model was also compared to substitution of zero using real qPCR data obtained from fecal samples spiked with known quantities of the tetracycline resistance gene *tetL*. An environmental isolate of *Enterococcus faecium* possessing the *tetL* gene was collected from a bovine fecal sample and grown overnight in TSA plus tryptose. Both genomic and plasmid borne variants of this gene have been detected in *E. faecium* [36]. The number of colony forming units/ml of pure cell culture was determined by plate counts of 10-fold serial dilutions, and the number of *tetL* gene copies per CFU was determined by running qPCR on DNA extracted from the cell culture. Fecal samples were then spiked with the serial dilutions of the cell culture. DNA was extracted three separate times from each spiked fecal sample using a previously published protocol [37]. Standards were created by extracting DNA from the same 10-fold serial dilutions of pure cell culture. The quantities of *tetL*/µl of DNA extract in each sample were estimated in triplicate by qPCR. The mean *tetL* quantity/µl of each sample was back calculated to log_10_ gene quantity per gram of feces, using the hierarchical model and manually with censored observations (<10 copies/µl) replaced by 0. Because there were no covariates associated with these data, Tobit regression was not evaluated on this dataset.

## Results

### Simulated Data

**Figure 1:**
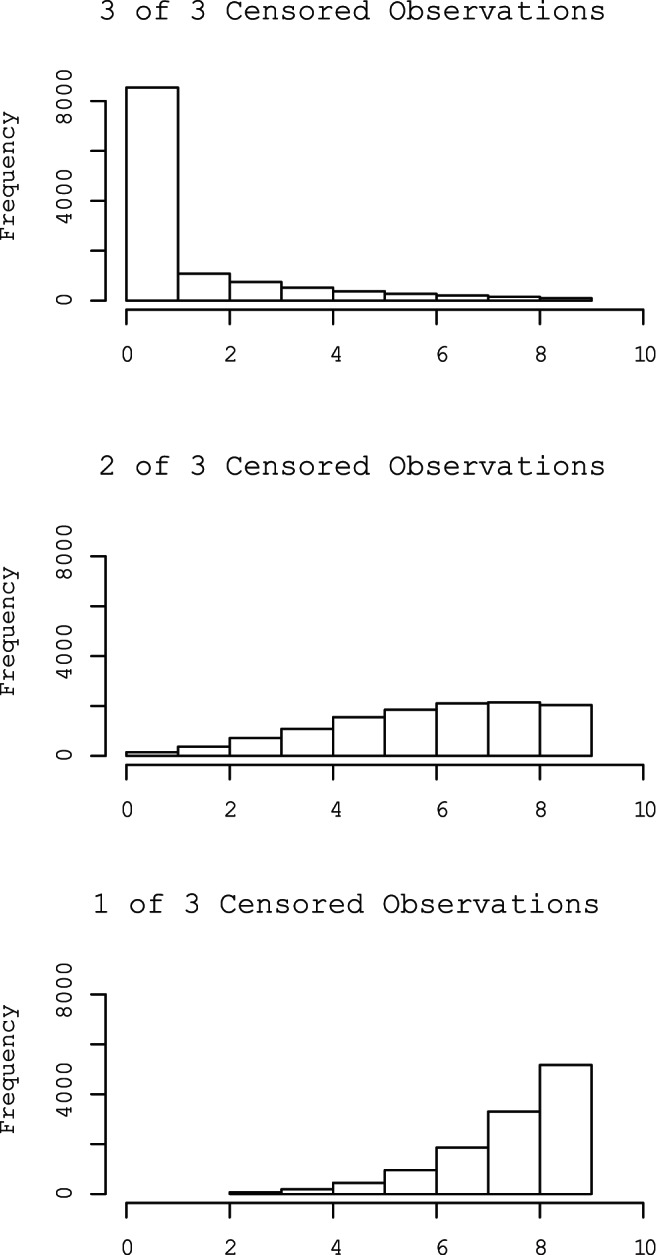
Posterior densities of three censored observations. The true sample averages from left to right were 0, 8.3, and 14.3 gene copies.

For each simulated dataset, the model estimated posterior densities for each sample replicate that was below 10 gene copies, using information present in uncensored observations. For observations that were part of a sample with three out of three censored observations, the mass of the posterior densities were centered over 0 with posterior means that were less than 1 gene copy (Figure 1). For observations that were part of a sample with two of three censored observations, the densities were more evenly dispersed, with posterior means of approximately 6 gene copies. For observations that came from samples with only one of three censored observations, the densities were centered toward the maximum of the range below the censoring limit, with posterior means of approximately 8 gene copies.

**Figure 2:**
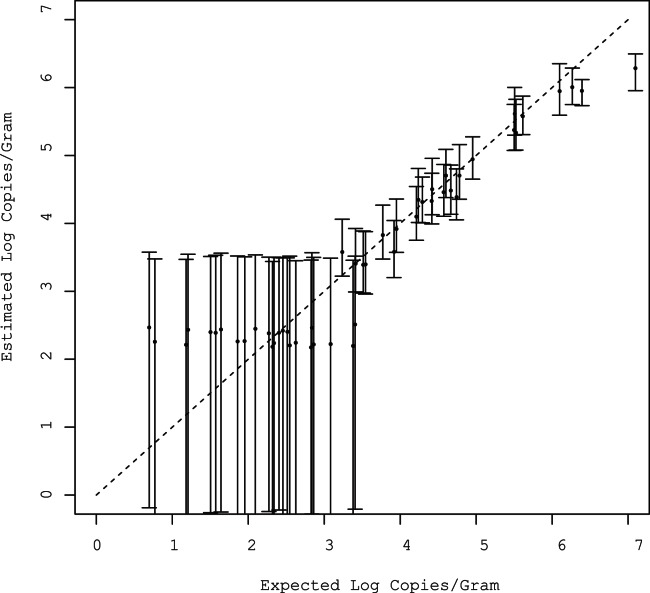
Posterior means and 95% credible intervals of log gene quantity versus the true value for one simulated data set with 50 observations.

To illustrate how the model estimated the log_10_ gene copy per gram of sample, the posterior means and 95% confidence intervals were plotted against the true values for one simulated dataset with parameters *β0* = 3, *β1* = 0.5, *σ* = 1.5 (Figure 2). For readability, a small sample size of 50 observations was used in this example; however, these results are typical of the simulations that were run with larger sample sizes and varying parameters. For samples with 0, 1, or 2 censored observations, the 95% CI’s are very narrow. For samples with 3 censored observations the 95% CI’s are very wide but still include the true values for all but the most extreme values of simulated data. In this example, samples with 3 censored observations had standard deviations approximately 0.8 log_10_ larger than samples with 0, 1, or 2 censored observations. The difference in precision of estimates among samples with 0, 1, or 2 censored triplicates was small. For example, the difference in the standard deviation between a sample with no censored observations and a sample with a similar true log_10_ gene quantity and two censored triplicates was approximately 0.02 log_10_.

The regression parameter estimates for the intercept, *β0*, represented the average log_10_ gene quantity per gram for samples that were not associated with the binary risk factor, with true values in each scenario ranging from 1.0–3.0 log_10_ gene copies. The estimates for *β1* represented the average increase in log_10_ gene quantity per gram for samples that were associated with the risk factor, with true values of 0.5 log_10_ in every scenario. For all three scenarios, the hierarchical model overestimated *β0* by 0.15–0.20 log_10_ gene copies per gram (Table 2). The degree of overestimation increased as the amount of censoring increased. The model underestimated the effect of the risk factor, *β1,* by −0.03 to −0.05 log_10_ gene copies per gram with the amount of underestimation decreasing as the degree of censoring increased. The model performed well in its estimation of *β1* in all three scenarios; the 95% credible intervals for *β1* included the true value in 93%–95% of the iterations. However, the 95% credible intervals for *β0* included the true value in only 72% to 85% of iterations.

**Table 2:** Bias, mean square error, and percent coverage of 95% confidence intervals of regression parameters estimated by the hierarchical model, by Tobit regression, and by linear regression with censored observations substituted with a value that approximates 0 on the log scale.

Scenario 1 (*β0* = 3)	Bias	MSE	95% CI coverage
Hierarchical Model			
*β0*	0.15	0.03	72%
*β1*	−0.05	0.02	95%
Tobit Model			
*β0*	0.13	0.03	75%
*β1*	−0.04	0.02	95%
Substitute 0.00325*			
*β0*	−0.23	0.06	45%
*β1*	−0.06	0.02	95%
**Scenario 2 (** ***β0*** ** = 2)**			
Hierarchical Model			
*β0*	0.19	0.06	79%
*β1*	−0.03	0.03	94%
Tobit Model			
*β0*	0.29	0.11	52%
*β1*	−0.07	0.03	93%
Substitute 0.00325*			
*β0*	−0.30	0.10	21%
*β1*	0.02	0.02	96%
**Scenario 3 (** ***β0*** ** = 1)**			
Hierarchical Model			
*β0*	0.20	0.21	85%
*β1*	−0.03	0.06	93%
Tobit Model			
*β0*	0.46	0.35	64%
*β1*	−0.06	0.05	93%
Substitute 0.00325*			
*β0*	−0.15	0.03	63%
*β1*	−0.11	0.03	87%

* The log of zero is undefined. To approximate an average gene quantity of 0 per sample, a value was substituted that, under the assumption of 65% extraction efficiency, back calculates to 1 gene copy per gram or 0 log_10_ copies.

The true values of the regression parameters were *β1* = 0.5, *σ* = 1.5, and *β0* = 3, 2, or 1.

**Figure 3:**
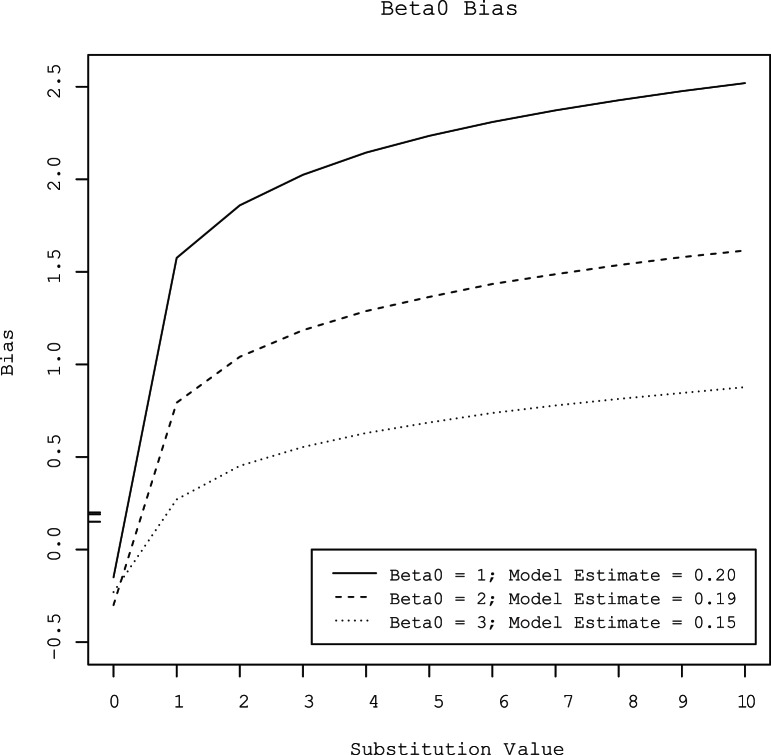
Bias of the intercept (*β0*) estimated by linear regression with censored observations substituted with values that ranged from 0 to 10 at three different levels of gene quantity (approximately 3, 2, and l log_10_ gene copies per gram). For comparison, the biases of the intercepts estimated by the hierarchical model at the three concentrations are provided in the legend and are shown as tick marks on the y-axis.

**Figure 4:**
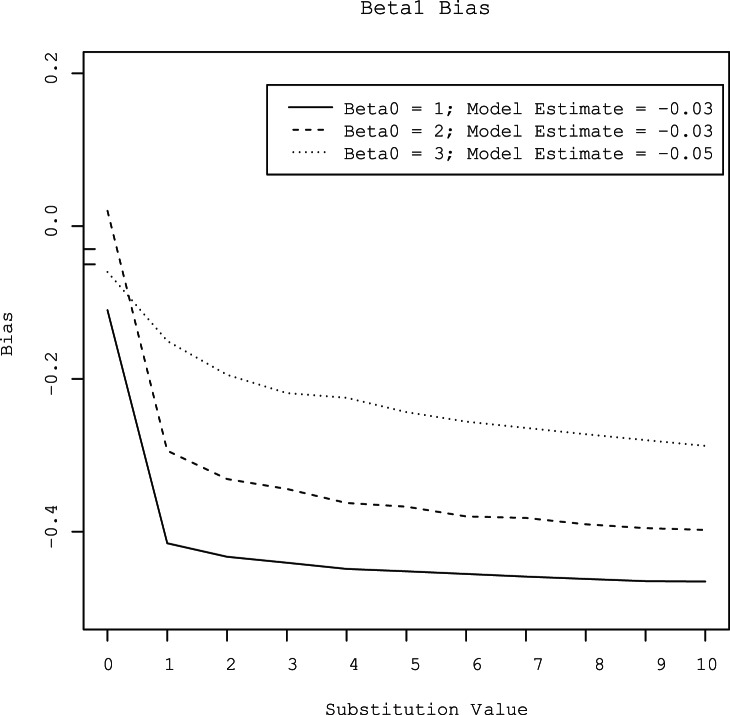
Bias of the generic risk factor (*β1*) estimated by a linear regression model with censored observations substituted with values that varied from 0 to 10, at three different levels of gene quantity (approximately 3, 2, and l log_10_ gene copies per gram). For comparison, the biases of the intercepts estimated by the hierarchical model at the three concentrations are provided in the legend and are shown as tick marks on the y-axis.

**Figure 5:**
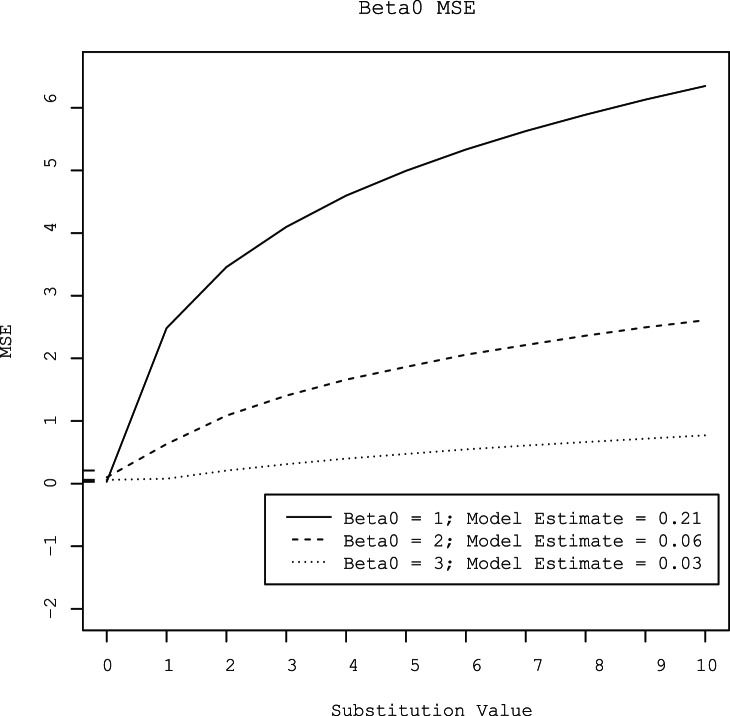
Mean square error (MSE) of the intercept (*β0*) estimated by linear regression with censored observations substituted with values that varied from 0 to 10 at three different levels of gene quantity (approximately 3, 2, and l log_10_ gene copies per gram). For comparison, the MSE’s of the intercepts estimated by the hierarchical model at the three concentrations are provided in the legend and are shown as tick marks on the y-axis.

**Figure 6:**
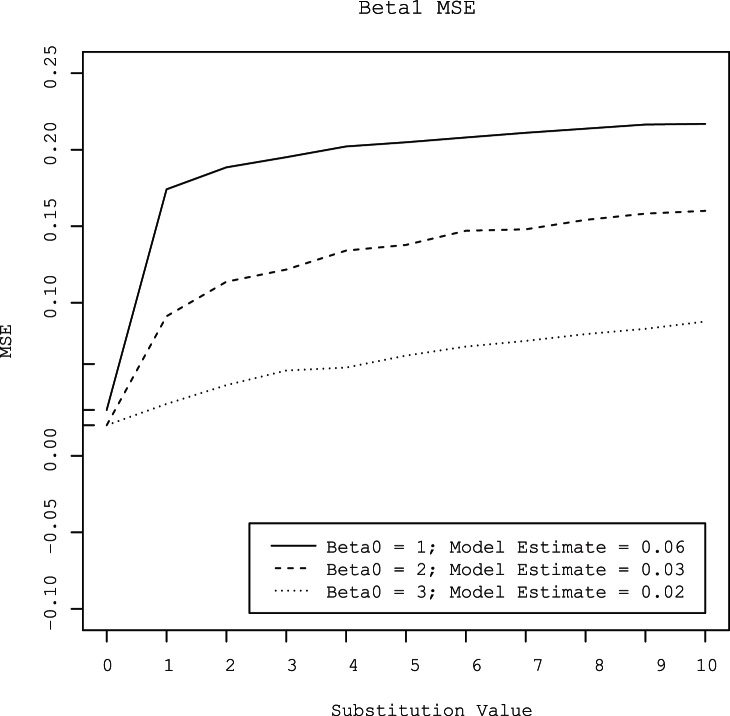
Mean square error (MSE) of the generic risk factor (*β1*) estimated by linear regression with censored observations substituted with values that varied from 0 to 10, at three different levels of gene quantity (approximately 3, 2, and l log_10_ gene copies per gram). For comparison, the MSE’s of the intercepts estimated by the hierarchical model at the three concentrations are provided in the legend and are shown as tick marks on the y-axis.

Substitution of censored observations with values ranging from 1 to 10 overestimated the value of *β0* by 0.27 to 2.5 log_10_ gene copies per gram (Figure 3). The amount of bias increased with the level of censoring. At all three levels of censoring, the degree of bias increased as the substituted value increased. Conversely, substitution of censored observations with values ranging from 1 to 10 underestimated the value of *β1* by 0.15 to 0.46 log_10_ gene copies per gram (Figure 4). As with *β0*, the amount of bias increased as the substituted value increased and as the degree of censoring increased. The MSE’s of parameter estimates also increased with increasing substitution value and increasing degree of censoring (Figures 5 and 6).

Substitution of a value approximating 0 copies resulted in *β0* values that were underestimated by a range of −0.15 to −0.30 (Table 2). The estimates of *β1* were also underestimated by −0.02 to −0.11 log_10_ gene copies. Coverage of the 95% confidence intervals of *β1* was good (95%–96%) except for scenario three when the coverage dropped to 87%. As with the hierarchical model, 95% CI coverage for *β0* was inadequate for all three scenarios (21%–63%). Of all the substitution values that were evaluated, a value approximating 0 was the only one the produced results that were comparable to the hierarchical model. For scenarios 1 and 2, the hierarchical model performed equally well as substitution of a value approximating 0 in estimation of *β1* (Table 2). Both approaches produced similar biases, MSE’s and 95% CI coverage. For scenario 3, the bias of the substitution of 0 approach increased and the 95% CI coverage dropped below acceptable levels. This was likely due to the fact that the proportion of observations that were censored was so high that there was not enough information in the scenario 3 datasets to adequately estimate *β1*. Both the hierarchical model and the substitution of zero method produced inadequate estimates of *β0* in all three scenarios. However, the hierarchical model performed slightly better than the substitution of zero method for scenarios 1 and 2 while the reverse was true for scenario 3.

The Tobit regression model produced results in Scenario 1 that were very similar to the hierarchical model; *β0* was over estimated by 0.13 and *β1* was underestimated by 0.04 and the MSE’s of the two approaches were equal (Table 2). In Scenarios 2 and 3 the hierarchical model outperformed Tobit regression.

When the value of the generic risk factor was reduced to from 0.5 to 0.1 (with *β0* = 3 and *σ* = 1.5), the hierarchical model produced the least biased estimates of *β0* and *β1*; however, the model also produced larger MSE’s for both parameters compared to Tobit regression (Table 3). The coverage of the 95% CI’s was better for the hierarchical model than for Tobit regression. Substitution of 0 considerably underestimated the value of *β0* and overestimated the value of *β1* by a larger percentage of the true value than was underestimated by the other two methods.

**Table 3:** Bias, mean square error, and percent coverage of 95% confidence intervals of regression parameters estimated by three methods: the hierarchical model, Tobit regression, and linear regression with censored observations substituted with a value that approximates 0 on the log scale.

Parameters: *β0* = 3, *β1* = 0.1, *σ* = 1.5	Bias	MSE	95% CI coverage
Hierarchical Model			
*β0*	0.003	0.24	94%
*β1*	−0.009	0.16	94%
Tobit Model			
*β0*	0.14	0.04	69%
*β1*	−0.007	0.02	94%
Substitute 0.00325*			
*β0*	−1.19	1.43	0%
*β1*	0.03	0.03	98%

The true values of the estimated parameters were *β0* = 3, *β1* = 0.1, and *σ* = 1.5.

### Antibiotic Resistance Gene Data

Estimation by qPCR of *tetL* gene quantity in the pure cell culture indicated that there were six gene copies per CFU. Based on these estimates, fecal samples were spiked with serial dilutions of *tetL* ranging from 6×10^7^ down to 6×10^2^ gene copies per gram of feces. DNA was extracted three times from each spiked fecal sample resulting in 18 fecal samples, each of which was run in triplicate, yielding 54 total observations that ranged from <10 copies to approximately 5.7×10^4^ gene copies per µl of DNA. The range of the standard curve was 1×10^7^ to 1×10^1^ gene copies per µl of DNA (efficiency = 96%, R^2^ = 0.999).

**Figure 7:**
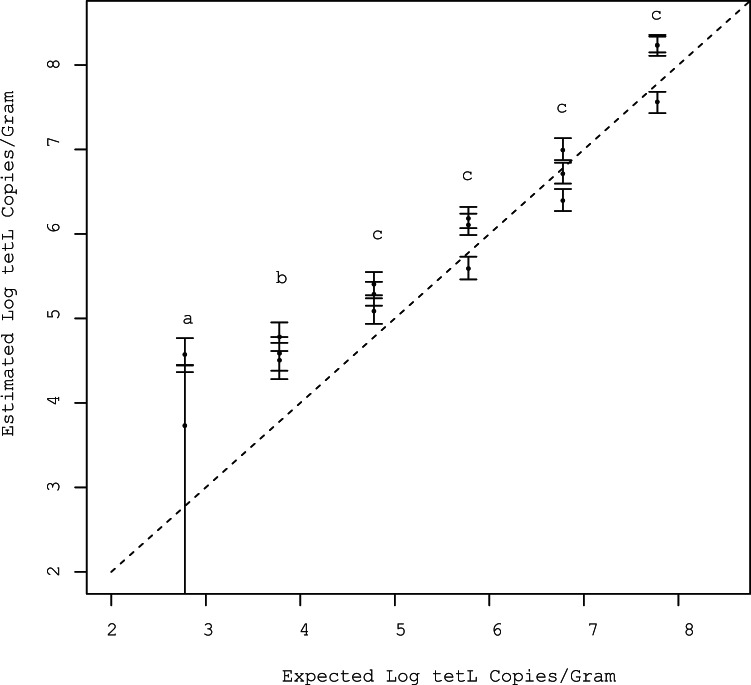
Posterior means and 95% credible intervals of log *tetL* quantity per gram versus the expected log *tetL* quantity per gram of spiked fecal samples. Observations denoted by the letter (a) had 8 of 9 censored observations, (b) had 4 of 9 censored, and (c) had 0 censored observations.

Overall, 12 of the 54 (22%) observations were censored. At the four highest concentrations there were no censored observations and the estimated sample mean quantities were within approximately 3-fold of the expected value (Figure 7). At the two lowest concentrations, five out of six samples had at least one censored observation. Two of the three samples spiked with 6×10^3^ gene copies had two censored triplicates while the third sample had no censored triplicates. Two of the three samples spiked with 6×10^2^ gene copies both had all three triplicates censored, while the third sample had two of three triplicates censored. The estimated mean sample gene quantities for samples with censored observations were overestimated by all three methods except for samples that had all three triplicates censored; the approach using a substitution of zero for censored observations underestimated the mean gene quantities in this scenario. The hierarchical model performed the best of the three approaches; the MSE of the estimates produced by the hierarchical model (0.526) was substantially lower than substitution of zero (1.16).

## Discussion

The performance of the hierarchical model was approximately the same across all three simulated scenarios; the estimates of the regression coefficient for the risk factor were close to the true value and the regression intercepts were overestimated in every scenario. Substitution of zero also produced accurate estimates of the risk factor coefficient for scenarios 1 and 2, but in scenario 3 when the level of censoring was extremely high, substitution of zero was no longer able to adequately estimate the risk factor coefficient. Tobit regression produced results that were comparable to the hierarchical model in Scenario 1, with the least censoring but was outperformed by the model in the other two scenarios. As with the hierarchical model, substitution of zero inadequately estimated the intercept but, in contrast to the hierarchical model, the intercept was underestimated. For most studies that investigate the relationship between gene quantity and a risk factor, the regression coefficient for the risk factor is the parameter that is of primary interest. Therefore, it is encouraging that, of the three approaches, the hierarchical model provided the most accurate estimates of the risk factor effect overall.

When applied to spiked fecal samples of known *tetL* quantity, the model overestimated the mean quantity of samples. Substitution of zero overestimated sample means when at least one replicate was uncensored but underestimated sample means when all three sample replicates were censored. The model was developed for use on data sets with moderate to high degrees of censoring, typically higher than the level censoring in the spiked *tetL* experiments. Nevertheless, the model still yielded a lower MSE than substitution of zero.

It was observed that the model underestimated the true simulated values when gene quantity was greater than 4 log_10_ per gram (Figure 1). However, the 95% CI’s of the posterior estimates continued to include the true value for large quantity samples except those greater than approximately 6 log_10_ per gram. This decline in performance for high quantity samples is most likely due to invalidation of the Central Limit Theorem, which provides the basis for the use of the Poisson distribution to approximate a binomial distribution [38]. The Poisson approximation to the binomial is generally described as appropriate when n is very large and p is very small and when n*p<10. This condition is true when the log_10_ gene quantity is less than approximately 3.5 (i.e. 10^3.5^ * 0.65 * 0.005 = 10.2). For the data sets used in these simulations the percentage of large quantity samples was small and not likely to affect the regression parameter estimates. The 95% CI’s of estimated log_10_ gene quantity failed to include the true value only for a small percentage of samples with greater than approximately 6 log_10_ copies.

In our experience, it is common to measure some antibiotic resistance genes by qPCR at levels greater than 6 log_10_ per gram. With these high quantity genes, the amount of censoring is very low if not completely absent and special analytical methods are not necessary. For other genes, the amount of censoring can be very high, similar to the simulated data in this study. For these low quantity genes, samples with greater than 6 log_10_ gene copies are relatively rare. In this situation, using the Poisson approximation to the binomial should be appropriate.

Past studies that have used qPCR to measure antibiotic resistance gene quantities in agricultural samples often do not provide the LOQ of their assays, nor do they discuss whether or not censoring was present in their data sets [17]−[25]. Therefore it is unknown if or how these studies dealt with censored observations. It is conceivable that censoring was variable depending on the gene being quantified; in our experience, some of the genes quantified in these studies range from very high to extremely low quantities. If high degrees of censoring were present in these studies and censored observations were replaced by substitution of a fixed value or were excluded, the results may have been biased.

### Conclusions

The general consensus for using qPCR to measure low quantity genes is that the number of replicates should be increased to improve the precision of estimates. For large studies this is not always feasible. Additionally, in epidemiologic studies involving qPCR, the main objective is often to measure an association between gene quantity and a risk factor in a population. In this case, precise estimation of gene quantity in individual samples is secondary to estimation of population parameters. Under the assumptions stated in the methods, the hierarchical model developed in this study provides a more accurate estimation of population parameters for moderately to highly censored qPCR data than do substitution of values close to 0 or Tobit regression. The use of a hierarchical model and MCMC methods has the added advantage of being able to accommodate additional levels to account for more complicated study designs that are common in epidemiology such as longitudinal studies with repeated measures within individuals or multilevel data such as animals clustered within herds.

## Supporting Information

Text S1 R code for simulations.
(DOCX)
