# Supplementary material for: Estimation of Low Quantity Genes: A Hierarchical Model for Analyzing Censored Quantitative Real-Time PCR Data
Source: PLoS One. 2013 May 31;8(5):e64900. doi: 10.1371/journal.pone.0064900 (PMC3669010; doi:10.1371/journal.pone.0064900)
Supplement: Text S1 — R code for simulations. (DOCX) [file pone.0064900.s001.docx]

**APPENDIX**

######################

# R code for simulations #

######################

library (R2WinBUGS)

# generate 500 simulated data sets of 500 samples each

n.fake<-500

n=500

# place holders for outputs

b0.mean<-rep(NA, n.fake)

b1.mean<-rep(NA, n.fake)

sigma.mean<-rep(NA, n.fake)

# Loop for each of the 500 simulations

for (s in 1:n.fake){

tx=rbinom(n,1,0.5) # tx is the generic risk factor

m=3+0.5*tx

y1=rnorm(n,m,1.5) # generates log sample quantity per gram

q=0.65

y2=q*10^y1 # transform quantity from the log scale

y3=round(y2) # round quantity

y3[y3>99999999]<-99999999

# capping y3 at high value prevents occasional errors in rmultinom function

########################################################################

# The following code converts the vectors of gene quantity per sample and the risk factor

# into a matrix of gene quantity per 5, 5, 5, and 985 microliters of DNA extract, a sample

# ID variable, a risk factor variable (tx).

########################################################################

dir=function(p){ g=rgamma(length(p),shape=p,scale=1); g/sum(g)}

y4=matrix(rep(0,n*3),n,3)

for(i in 1:n){y4[i,1:3]=rmultinom(1,y3[i],dir(c(5,5,5,985)))[1:3]}

data<-cbind(y4, tx)

data<-as.data.frame(data)

data<-reshape(data, direction="long", varying=(1:3), v.names="well")

w<-data$well

t<-data$tx

sample<-data$id

z <- w

z[z<10] <- NA # convert values below LOQ to missing

w[w<10] <- 9

data<-data.frame(sample, w, z, tx)

sortdata <- data[order(sample) , ]

sample<-sortdata$sample

w<-sortdata$w

z<-sortdata$z

t<-sortdata$t

J<-1500

I<-500

#########################################################

# Specify the data, initial values, and model to call WinBUGS from R

#########################################################

sim.data <- list(I=I, J=J, z=z, w=w, sample=sample, tx=tx)

sim.inits <- function (){

list (tau=1, b0=3, b1=1)

}

sim.parameters <- c("sigma", "b0", "b1", "y", "z")

sim.fake <- bugs(sim.data, sim.inits, sim.parameters, "sim_model.bug",

n.chains=1, n.iter=5000, n.burnin=1000, n.thin=1, n.sims=4000,

bugs.directory="c:/Program Files/WinBUGS14/",

working.directory="Insert path of WinBUGS working directory ",

clearWD=FALSE, DIC=FALSE, debug=FALSE)

# Store outputs from each simulation

attach.bugs(sim.fake)

b0.mean[s]<-mean(b0)

b1.mean[s]<-mean(b1)

sigma.mean[s]<-mean(sigma)

}

##################################################################

# WinBUGS code kept in working directory as a text file with .bug extension

##################################################################

model{

for(j in 1:J){

upperlimit[j]<-10000000+(-10000000+9)*equals(w[j], 9)

z[j] ~ dpois(lambda[sample[j]]) I( , upperlimit[j])

}

for(i in 1:I){

lambda[i] <- q[i]*pow(10,y[i])*p[i]

p[i] ~ dbeta(5,995)

q[i] <- 0.65

y[i] ~ dnorm(mu[i],tau)

mu[i] <- b0+b1*tx[i]

}

tau ~ dgamma(0.001, 0.001)

sigma <- 1/sqrt(tau)

b0 ~ dnorm(0,0.001)

b1 ~ dnorm(0,0.001)

}
